# Heat shock factor 1 confers resistance to lapatinib in ERBB2-positive breast cancer cells

**DOI:** 10.1038/s41419-018-0691-x

**Published:** 2018-05-24

**Authors:** Alisha Yallowitz, Amr Ghaleb, Lucas Garcia, Evguenia M. Alexandrova, Natalia Marchenko

**Affiliations:** 1 Department of Pathology, Stony Brook University, Stony Brook, NY 11794-8691 USA; 2 Weill Cornell Medicine, 1300 York Avenue, LC-902, New York, NY 10065 USA

## Abstract

Despite success of ERBB2-targeted therapies such as lapatinib, resistance remains a major clinical concern. Multiple compensatory receptor tyrosine kinase (RTK) pathways are known to contribute to lapatinib resistance. The heterogeneity of these adaptive responses is a significant hurdle for finding most effective combinatorial treatments. The goal of this study was to identify a unifying molecular mechanism whose targeting could help prevent and/or overcome lapatinib resistance. Using the MMTV-ERBB2;mutant p53 (R175H) in vivo mouse model of ERBB2-positive breast cancer, together with mouse and human cell lines, we compared lapatinib-resistant vs. lapatinib-sensitive tumor cells biochemically and by kinome arrays and evaluated their viability in response to a variety of compounds affecting heat shock response. We found that multiple adaptive RTKs are activated in lapatinib-resistant cells in vivo, some of which have been previously described (Axl, MET) and some were novel (PDGFRα, PDGFRβ, VEGFR1, MUSK, NFGR). Strikingly, all lapatinib-resistant cells show chronically activated HSF1 and its transcriptional targets, heat shock proteins (HSPs), and, as a result, superior tolerance to proteotoxic stress. Importantly, lapatinib-resistant tumors and cells retained sensitivity to Hsp90 and HSF1 inhibitors, both in vitro and in vivo, thus providing a unifying and actionable therapeutic node. Indeed, HSF1 inhibition simultaneously downregulated ERBB2, adaptive RTKs and mutant p53, and its combination with lapatinib prevented development of lapatinib resistance in vitro. Thus, the kinome adaptation in lapatinib-resistant ERBB2-positive breast cancer cells is governed, at least in part, by HSF1-mediated heat shock pathway, providing a novel potential intervention strategy to combat resistance.

## Introduction

Human epidermal growth factor receptor 2 (Her2, ERBB2) is overexpressed in about 25% of sporadic human breast cancer cases, which correlates with poor prognosis^1^. Several ERBB2-targeted therapies are currently available that improve patients’ outcomes, including a dual ERBB2/EGFR kinase inhibitor lapatinib^2^. However, acquired resistance to lapatinib remains a major concern for its clinical utilization.

Multiple mechanisms of lapatinib resistance are described in the literature. They primarily involve compensatory activation of receptor tyrosine kinases (RTKs), such as ERBB3, IGF1R, MET, FGFR2, FAK, Axl, as well as other mechanisms^2^. Importantly, not a single, but multiple RTKs have been shown to be activated in response to lapatinib^3^. Also, the substantial heterogeneity among adaptive RTKs exists in different cell lines in response to lapatinib^3^. This represents a major hurdle for the development of successful combinatorial strategies to reverse and/or prevent lapatinib resistance. Hence, identification and targeting of an upstream effector governing the kinome adaption in response to ERBB2 inhibition would help to overcome this clinical dilemma.

Our previous studies identified heat shock factor 1 (HSF1) as a key effector of ERBB2 signaling^4–6^. HSF1 is a transcription factor that controls a broad spectrum of pro-survival events essential for protecting cells from proteotoxic stress, which is caused by the accumulation of misfolded proteins in cancer cells. HSF1 activates transcription of genes that regulate protein homeostasis, including heat shock proteins (HSPs), Hsp27, Hsp70, and Hsp90^7^, as well as supports other oncogenic processes such as cell cycle regulation, metabolism, adhesion, and protein translation^8, 9^. The impact of HSF1 on ERBB2-driven mammary tumorigenesis was unequivocally proven by in vivo studies. The genetic ablation of HSF1 suppresses mammary hyperplasia and reduces tumorigenesis in ERBB2 transgenic mice^10^. Consistently, the stability of ERBB2 protein is shown to be maintained by transcriptional targets of HSF1: Hsp70, Hsp90^11^, and Hsp27^7^.

Mutations in the *TP53* gene (mutp53) are the most frequent genetic events in ERBB2-positive breast cancer (72%)^12^ and correlate with poor patient outcomes^13^. To recapitulate human ERBB2-positive breast cancer in mice, we previously generated a novel mouse model that combines activated ERBB2 (MMTV-ERBB2 allele^14^) with the mutp53 allele R172H corresponding to human hotspot mutp53 allele R175H^12^. We found that mutp53 accelerates ERBB2-driven mammary tumorigenesis^15^. The underlying molecular mechanism is a mutp53-driven oncogenic feed-forward loop governing a superior survival of cancer cells. We found that mutp53, through enhanced recycling and/or stability of ERBB2/EGFR, augments MAPK and PI3K signaling, leading to transcriptional phospho-activation of HSF1 at Ser326. Furthermore, mutp53 directly interacts with phospho-activated HSF1 and facilitates its binding to DNA-response elements, thereby stimulating transcription of HSPs^5^. In turn, HSPs more potently stabilize their oncogenic clients ERBB2, EGFR, mutp53, HSF1, thus reinforcing tumor development^5^. Consistently, we found that lapatinib not only suppresses tumor progression, but does so, at least in part, via inactivation of HSF1^15^. Furthermore, the interception of the ERBB2-HSF1-mutp53 feed-forward loop by lapatinib destabilizes mutp53 protein in Hsp90-dependent and Mdm2-dependent manner^4^. Since mutp53 ablation has been shown to have therapeutic effects in vivo^16^, it is possible that mutp53 destabilization by lapatinib contributes to its anti-cancer activity.

In the present study, we identified HSF1 as an important upstream node responsible for the kinome adaptation of lapatinib-resistant cells. We found that lapatinib-resistant cancer cells have enhanced HSF1 activity, a superior resistance to proteotoxic stress, and lose their ability to degrade mutp53 in response to lapatinib. In contrast, HSF1 inhibition blocks lapatinib-induced kinome adaption and prevents the development of lapatinib resistance. Our data suggest a mechanism-based rationale for the clinical utilization of HSF1 inhibitors for the treatment of lapatinib-resistant ERBB2-positive breast cancer and/or—in combination with lapatinib—to prevent development of lapatinib resistance.

## Results

### Generation and characterization of human and mouse lapatinib-resistant ERBB2-positive breast cancer cell lines

**Fig. 1:**
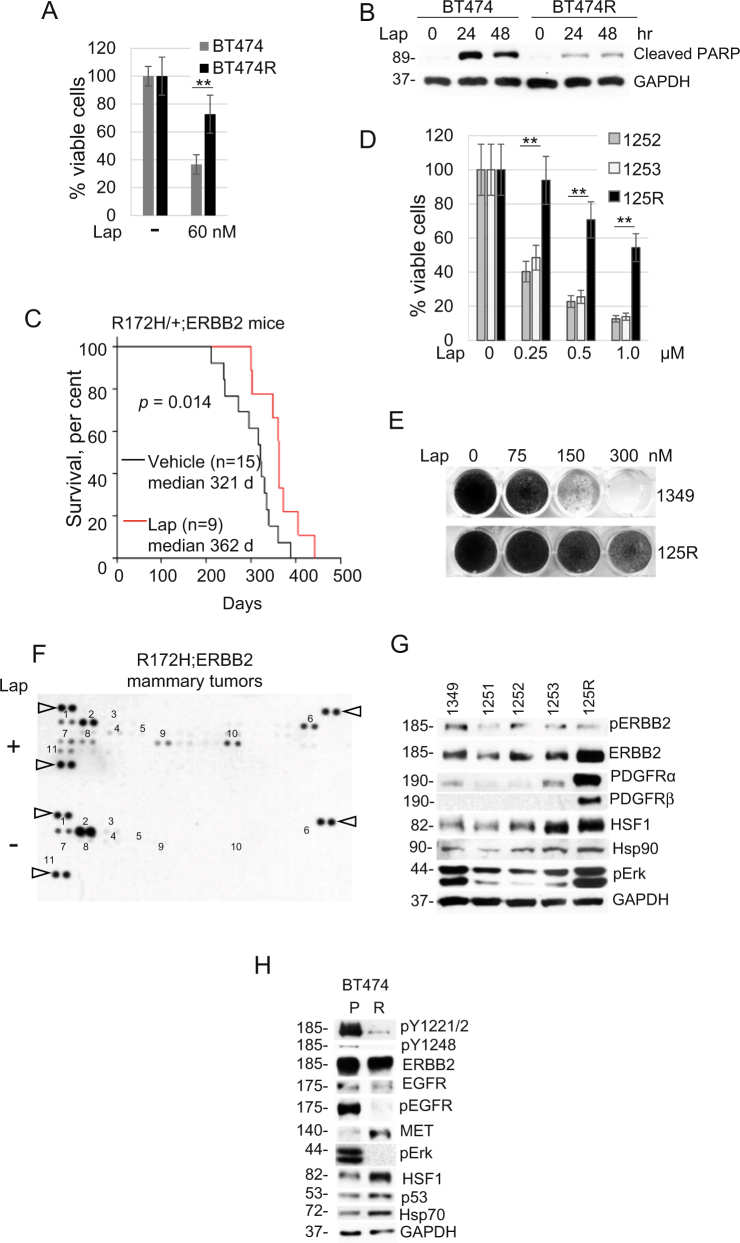
Generation and characterization of lapatinib-resistant human and mouse Her2-positive cancer cell lines. **a**, **b** Lapatinib-resistant human BT474R cells exhibit a two-fold increased viability after 48 h treatment with lapatinib (**a**) and a significantly decreased apoptosis after treatment with 300 nM lapatinib for indicated times (measured by cleaved PARP, **b**) compared to lapatinib-sensitive parental BT474 cells. **b** Western blot analysis, GAPDH is a loading control. One representative experiment out of two independent experiments (each performed in triplicate) is shown; **p < 0.01 for three technical replicas, Student’s *t*-test (**a**). **c** Tumor onset is significantly delayed in R172H/+;ERBB2 females treated with 75 mg/kg lapatinib three times a week starting at 8 weeks of age lifelong (red line) compared to vehicle-treated siblings (black line). Kaplan–Meier analysis, log rank statistics. **d**, **e** Murine primary cell lines established from lapatinib-sensitive mammary tumors (1252, 1253, 1349) and from a lapatinib-resistant mammary tumor (125R) maintain lapatinib sensitivity and lapatinib resistance in vitro, respectively. **d** Short-term cell viability assay (48 h). One representative experiment out of two independent experiments (each performed in triplicate) is shown; **p < 0.01 for three technical replicas, Student’s *t*-test. **e** Long-term colony formation assay (4 weeks). **f** Lapatinib induces activation of multiple adaptive RTKs in vivo. Representative images of the mouse Phospho-RTK array kit comparing lapatinib-treated (i.e., lapatinib-resistant, top) with vehicle-treated (i.e., lapatinib-sensitive, bottom) tumors. 1. EGFR*, 2. ERBB2*, 3. ERBB3, 4. PDGFRα*, 5. PDGFRβ*, 6. Axl*, 7. NGFR, 8. VEGFR1*, 9. MUSK*, 10. EPHA2*, 11. EPHB2. Known Hsp90 clients are marked with an asterisk. Triangles mark reference spots. **g**, **h** Murine lapatinib-resistant 125R cells show upregulated PDGFRα and PDGFRβ compared to murine lapatinib-sensitive cells 1349, 1251, 1252, 1253 (**g**), while human lapatinib-resistant BT474R cells have upregulated MET and downregulated ErbB2 and EGFR signaling (measured by phospho-ERBB2 pY1221/2 and pY1248, two top panels, phospho-EGFR and their effector phospho-Erk), compared to parental BT474 cells (**h**). Note that both human and mouse lapatinib-resistant cells have upregulated HSF1 and its transcriptional targets Hsp90 (**g**) and Hsp70 (**h**). Western blot analysis, constitutive heat shock protein 70 (Hsc70) and GAPDH served as a loading control

To gain the mechanistic insight into lapatinib resistance we utilized two complementary approaches: in vitro and in vivo. For in vitro studies, we continuously cultivated human ERBB2-positive BT474 breast cancer cells in the presence of increasing concentrations (100–300 nM) of lapatinib for 6 months. All selected lapatinib-resistant clones were combined and maintained as a pool, as previously described^3^. Lapatinib-resistant cells approximately doubled their viability compared to lapatinib-sensitive cells (Fig. 1a), which was associated with decreased apoptosis in the presence of lapatinib (Fig. 1b).

To investigate lapatinib resistance acquired in vivo, we utilized the previously described MMTV-ERBB2;R172H mouse model of ERBB2-positive breast cancer (“R172H/+;ERBB2” hereafter)^15^. At the age of mammary micro-lesions (8-weeks old), R172H/+;ERBB2 females were given lapatinib (75 mg/kg three times a week) or vehicle by oral gavage, lifelong. Consistent with human data, lapatinib shows a tendency to delay tumor onset (from 256 to 319 days, median onset, *p* = 0.091) and significantly extended overall survival (from 321 to 362 days, median survival, *p* = 0.014) compared to vehicle-treated mice (Fig. 1c). However, after initial response (Fig. 1c) mammary tumors acquired lapatinib resistance and started to exhibit growth kinetics similar to vehicle-treated tumors (Fig. 4a).

We established cell lines from both vehicle-treated (lapatinib-sensitive; 1349, 1347, 1251, 1252, 1253) and lapatinib-treated (lapatinib-resistant; 125R) mouse mammary tumors. In contrast to previous studies using human ERBB2-positive breast cancer cell lines^3^, our murine cell lines were derived from littermates, have an identical genetic background, the same mutation and acquired lapatinib resistance in vivo (with normal gland architecture, tumor microenvironment, immune system status), and therefore should better reflect the resistance mechanisms encountered in patients in the clinic. The short-term cell viability assay and the long-term colony formation assays both confirmed that the established cell lines continued to maintain their lapatinib resistance acquired in vivo (Fig. 1d, e).

To test for possible compensatory mechanisms induced in vivo, we performed the kinome profiling of 39 activated RTKs in lapatinib-treated vs. vehicle-treated tumors, respectively. In lapatinib-treated tumors we found expected downregulation of phospho-activated ERBB2 and EGFR and upregulation of multiple compensatory RTKs (Fig. 1f), including previously described Axl^2^ and novel RTKs, such as NFGR, MUSK, VEGFR1, PDGFRα, PDGFβ, EPHA2, and EPHB2 (Fig. 1f). These results suggest a robust kinome reprogramming and a switch to multiple alternative RTKs in lapatinib-resistant cells. Consistently, we observed enhanced phospho-Erk, a common downstream RTK effector, in lapatinib-resistant 125R murine cell line (Fig. 1g). We validated the arrays data by Western blot analysis of the cell lines established from murine mammary tumors. Consistent with the array, PDGFRα and PDGFRβ were upregulated in lapatinib-resistant 125R cells compared to lapatinib-sensitive cells (Fig. 1g). Interestingly, in human lapatinib-resistant BT474 PDGFRα and PDGFRβ were not upregulated, and, instead, MET was elevated (Fig. 1h). This difference likely reflects the heterogeneity in adaptive responses noted previously^3^.

Despite the distinct adaptive RTK response in mouse vs. human lapatinib-resistant cancer cells, notably, they share an important common feature, i.e., stabilized PDGFRα, PDGFRβ, and MET that are maintained by Hsp90 (https://www.picard.ch/downloads/Hsp90interactors.pdf). Therefore, we hypothesized that HSF1-mediated heat shock response is causative to the observed adaptive RTKs upregulation in lapatinib-resistant cells. Indeed, this link is supported by the fact that six out of eight RTKs upregulated in lapatinib-resistant mammary tumors—Axl, VEGFR1, MUSK, PDGFRβ, PDGFRα, EPHA2—are known Hsp90 clients (www.picard.ch/downloads/Hsp90interactors.pdf).

### Lapatinib-resistant breast cancer cells are resistant to proteotoxic stress

**Fig. 2:**
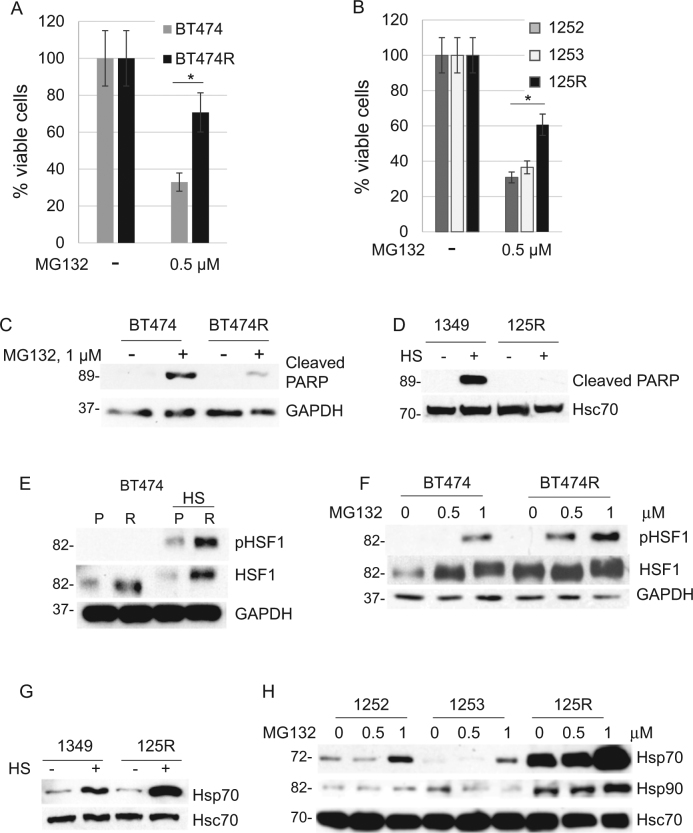
Lapatinib-resistant cells are protected from proteotoxic stress. **a**, **b** Lapatinib-resistant human BT474R cells (**a**) and mouse 125R cells (**b**) are more resistant to proteotoxic stress induced by the proteasome inhibitor MG132 (0.5 μM for 48 h) than their corresponding lapatinib-sensitive control cells. Cell viability assay. One representative experiment out of two independent experiments (each performed in triplicate) is shown; *p < 0.05 for three technical replicas, Student’s *t*-test. **c**–**e** Under proteotoxic stress induced by (**c**) the proteasome inhibitor MG132 (1 μM for 48 h) and (**d**) heat shock (43 C, 30 min, Western blot 48 h after) lapatinib-resistant human and murine cells have decreased apoptosis (cleaved PARP) and increased phospho-HSF1 (Ser326 **e**, **f**) compared to lapatinib-sensitive cells. Lapatinib-resistant murine 125R cells show upregulated heat shock protein Hsp70 to lapatinib-sensitive cells after proteotoxic stress induced by (**g**) heat shock (43 C, 30 min, Western blot 2 h after) and proteasome inhibitor MG132 (**h**). Western blot analysis. GAPDH and Hsc70 as a loading control

To test whether HSF1-induced heat shock response is involved in the kinome adaptation of lapatinib-resistant cells, we compared their viability under the proteotoxic stress condition with lapatinib-sensitive cells. We found both the cells that acquired lapatinib resistance in vitro (Fig. 2a) and in vivo (Fig. 2b) to be more resistant to the proteotoxic stress induced by the proteasome inhibitor MG132 (Fig. 2c) and heat shock (Fig. 2d), which correlated with reduced apoptosis measured by PARP cleavage (Fig. 2c, d).

HSF1 reveals its protective role under proteotoxic stress via transcriptional activation of HSPs by transcriptionally active pSer326-HSF1. Indeed, upon proteotoxic stress induced by heat shock (Fig. 2e) and proteasome inhibition (Fig. 2f) lapatinib-resistant BT474 cells show a higher level of pSer326-HSF1. Since pSer326-HSF1 antibodies are human specific, we tested activity of pHSF1 in murine lapatinib-resistant 125R cells by the level of HSF1 transcriptional targets Hsp70 and Hsp90, and again found their significant upregulation upon proteotoxic stress induced by heat shock (Fig. 2g) and proteasome inhibition (Fig. 2h). These data indicate that lapatinib resistance correlates with augmented HSF1 function, and, as a result, with a superior tolerance to proteotoxic stress.

### Lapatinib fails to modulate the ERBB2–HSF1–mutp53 axis in lapatinib-resistant breast cancer cells

**Fig. 3:**
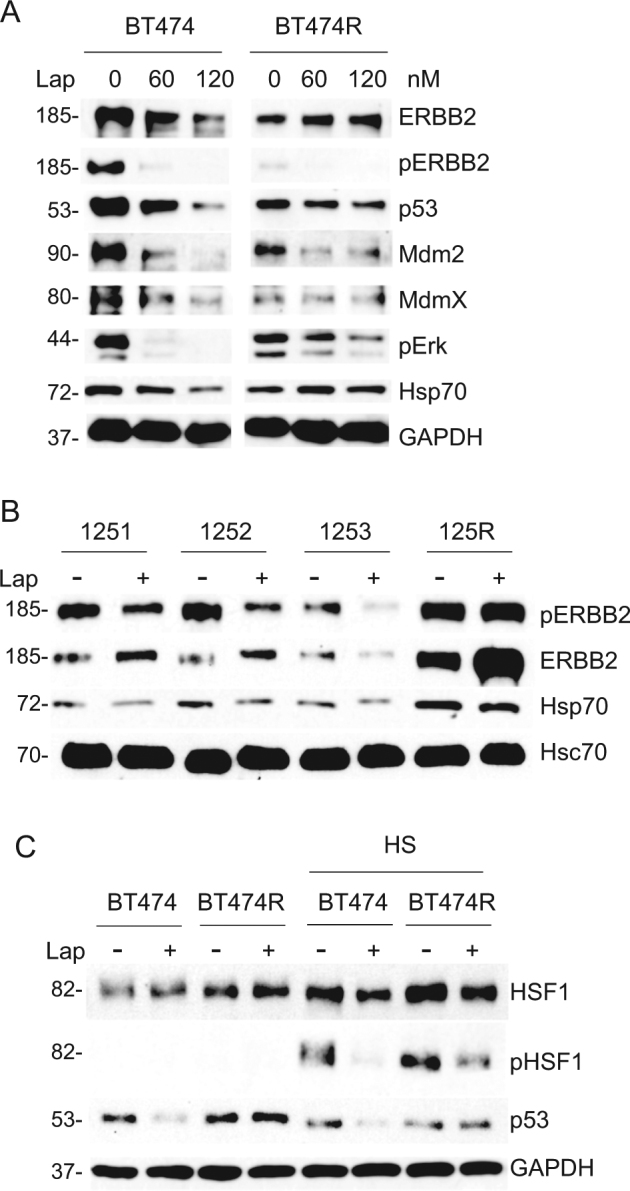
Lapatinib fails to modulate the ErbB2–HSF1–mutp53 axis in lapatinib-resistant cells. **a** Downregulation of HSF1, mutp53, and Mdm2 in lapatinib-sensitive parental BT474, but not in lapatinib-resistant BT474R cells, treated with indicated lapatinib concentrations for 24 h (**a**). **b** Lapatinib (300 nM, 48 h) blocks ERBB2 activation (measured by phospho-ERBB2) and its downstream effector Hsp70 in murine lapatinib-sensitive (1251, 1252, 1253), but not in lapatinib-resistant 125R cells. Hsc70 served as a loading control. **c** Lapatinib (300 nM, 24 h) blocks HSF1 activation (measured by phospho-Ser326) in human lapatinib-sensitive BT474, but not in lapatinib-resistant BT474R cells after heat shock (42°C, 30 min). Cells were pre-treated with lapatinib followed by heat shock. Western blot analyses. GAPDH as a loading control

Previously we showed that lapatinib destabilizes mutp53 via inhibition of HSF1 activity^4^. We now tested the effect of lapatinib on mutp53 levels in lapatinib-resistant BT474 cells (Fig. 3a) and found that lapatinib lost its ability to destabilize mutp53 even at higher doses (Fig. 3a), likely as a result of chronic HSF1 activity. Indeed, lapatinib did not suppress HSF1 transcriptional target Hsp70, compared to lapatinib-sensitive cells, and failed to induce auto-degradation of Mdm2 and its bona fide substrates MdmX and mutp53 (Fig. 3a). Since previous studies identified highly stabilized mutp53 protein as an essential pro-survival factor in cancer cells^17^, mutp53 depletion by lapatinib in lapatinib-sensitive cells could further enhance lapatinib’s efficiency, while unresponsive high levels of mutp53 in lapatinib-resistant cells might contribute to the resistance mechanism. Similarly, lapatinib inhibited ERBB2 signaling (measured by phospho-ERBB2) and Hsp70 levels in sensitive murine lines, but failed to do so in the lapatinib-resistant murine 125R cells (Fig. 3b).

Proteotoxic stress induced by heat shock leads to transcriptional activation of HSF1 by Ser326 phosphorylation in both lapatinib-sensitive and resistant BT474 cells. However, lapatinib prevents phospho-activation of HSF1 after heat shock only in lapatinib-sensitive cells (Fig. 3c, compare lanes 5 and 6), but not in lapatinib-resistant BT474 cells (Fig. 3c, compare lanes 7 and 8). Most likely, HSF1 lost its dependency on the ERBB2 signaling in lapatinib-resistant cells due to the switch to alternative RTKs and their downstream effectors like Erk and Akt^17, 18^, which reconstitutes HSF1 function and supports cells survival after ERBB2 inhibition^19^.

Altogether, these data reinforce that despite of the heterogeneity of adaptive responses, tumors acquire lapatinib resistance, at least in part, via unified HSF1-guided mechanism that feeds into stabilization of mutp53.

### Lapatinib-resistant breast cancer cells are sensitive to Hsp90 inhibition

**Fig. 4:**
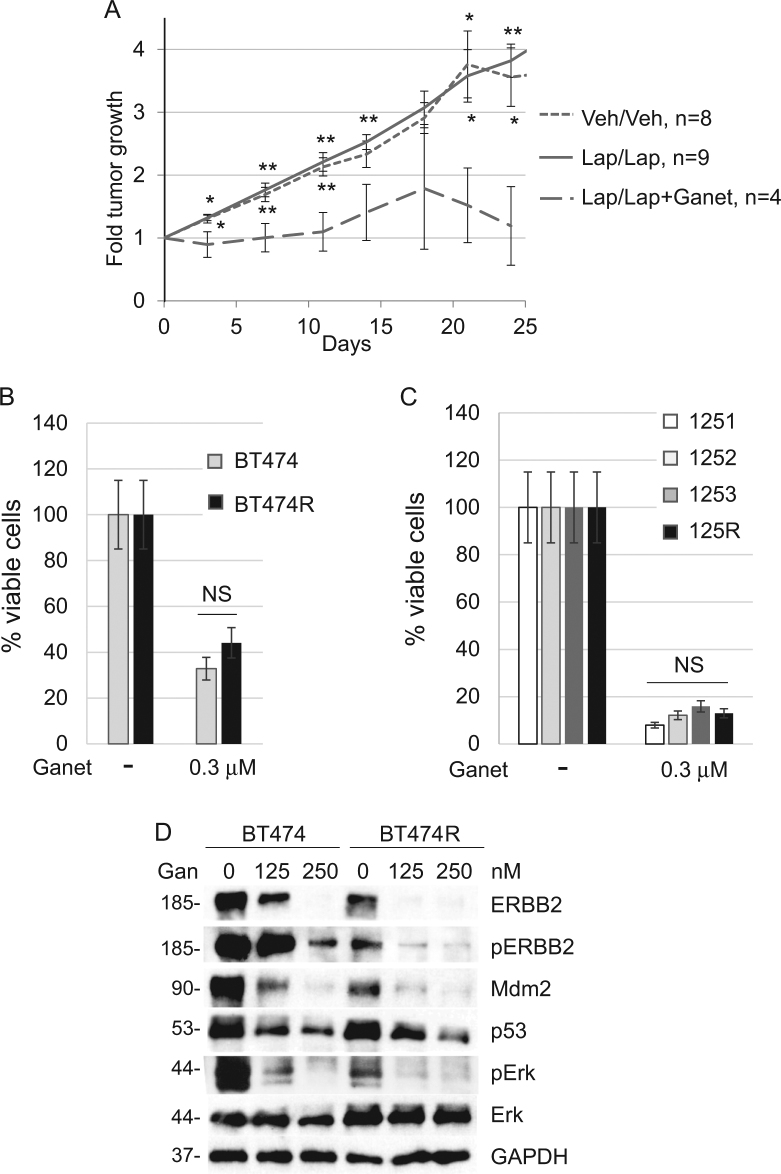
Lapatinib-resistant breast cancer cells are sensitive to Hsp90 inhibition. **a** R172H/+;ERBB2 female mice were treated either with vehicle or 50 mg/kg lapatinib (three times a week starting at 8 weeks of age) until tumors acquired lapatinib resistance, i.e., lapatinib no longer suppressed tumor growth. At this point, previously vehicle-treated mice were continued on vehicle (Veh/Veh), while previously lapatinib-treated mice continued to be treated with either lapatinib alone (Lap/Lap) or with lapatinib together with 50 mg/kg ganetespib once a week (Lap/Lap + Ganet), as described in Results. Note that the initial tumor size in all three groups was on average comparable. Tumor size was measured and plotted every 5 days. The treatment has been ended and mice were sacrificed when tumors in Veh/Veh and Lap/Lap arms reached the size of 3.5 cm^3^. Note that while lapatinib-resistant tumors grew similarly to untreated tumors (did not respond to lapatinib), addition of ganetespib significantly suppressed tumor growth (wide-dash line). *p < 0.05, **p < 0.01, Student’s *t*-test. Top asterisks compare the Lap/Lap + Ganet group with Lap/Lap, bottom asterisks compare the Lap/Lap + Ganet group with the Veh/Veh group. n number of independent tumors. **b**, **c** Lapatinib-sensitive human BT474 (**b**) and murine 125R (**c**) cells have similar sensitivity to ganetespib as their corresponding lapatinib-sensitive cells (BT474 and 1251, 1252, 1253, respectively). Cells were treated with DMSO or 0.3 µM ganetespib for 48 h, followed by the cell viability assay. One representative experiment out of two independent experiments (each performed in triplicate) is shown; NS non-significant. **d** Ganetespib (indicated concentrations, 24 h) inhibits ERBB2 signaling (measured by phospho-ERBB2 and phospho-Erk) and destabilizes mutp53 and Mdm2 in both, lapatinib-sensitive BT474 and lapatinib-resistant BT474R cells. Western blot analysis, GAPDH is a loading control

Since the majority of adaptive RTKs that we identified in vivo (Fig. 1f) are known Hsp90 clients, we hypothesized that lapatinib-resistant cells retain their sensitivity to Hsp90 inhibitors. To test this hypothesis, we used ganetespib, a new generation Hsp90 inhibitor, which is currently in several clinical trials^20^. First, we tested the effect of ganetespib in vivo, using R172H/+;ERBB2 mice with mammary tumors that have been previously treated with lapatinib until they acquired resistance, i.e., lapatinib no longer suppressed their growth. Starting with the same average tumor size in each group, we designated three groups of animals (Fig. 4a): (i) animals previously treated with vehicle were continued on vehicle (Veh/Veh); (ii) animals previously treated with lapatinib (i.e., lapatinib-resistant) were continued on lapatinib alone (75 mg/kg three times a week lifelong) (Lap/Lap); (iii) some animals previously treated with lapatinib (i.e., lapatinib-resistant) were continued on lapatinib (75 mg/kg three times a week lifelong) together with ganetespib (50 mg/kg once a week lifelong) (Lap/Lap + Gan).

Consistently with their lapatinib resistance, the tumors on lapatinib alone continued to grow fast, with the rate very similar to vehicle-treated tumors (Fig. 4a, solid vs. small-dash lines). In contrast, addition of ganetespib significantly suppressed growth of lapatinib-resistant tumors (Fig. 4a, wide-dash vs. small-dash lines). These data demonstrate that, despite lapatinib and ganetespib having overlapping targets (ERBB2, EGFR, mutp53), ganetespib overcomes lapatinib-resistant adaptive responses and efficiently curbs growth of lapatinib-resistant tumors in vivo. Consistently with these in vivo data, both human and mouse lapatinib-resistant cell lines were highly sensitive to ganetespib in vitro (Fig. 4b, c). As expected, ganetespib effectively inhibited ERBB2 signaling (measured by phospho-ERBB2) and—contrary to lapatinib—depleted mutp53 and Mdm2 in both lapatinib-sensitive and lapatinib-resistant cells (Fig. 4d). We speculate that ganetespib suppresses growth of lapatinib-resistant tumors via two complementary mechanisms: targeting of compensatory RTKs and release Mdm2 from the Hsp90 inhibitory complex, leading to mutp53 degradation.

### HSF1 inhibition targets mutp53 and ERBB2 for degradation and suppresses growth of lapatinib-resistant breast cancer cells

Although Hsp90 inhibition seems to be an effective strategy to overcome lapatinib resistance, it has significant limitations. Hsp90 inhibitors have been shown to activate HSF1-mediated heat shock response, which in the long run protects cancer cells from apoptosis^7^.

**Fig. 5:**
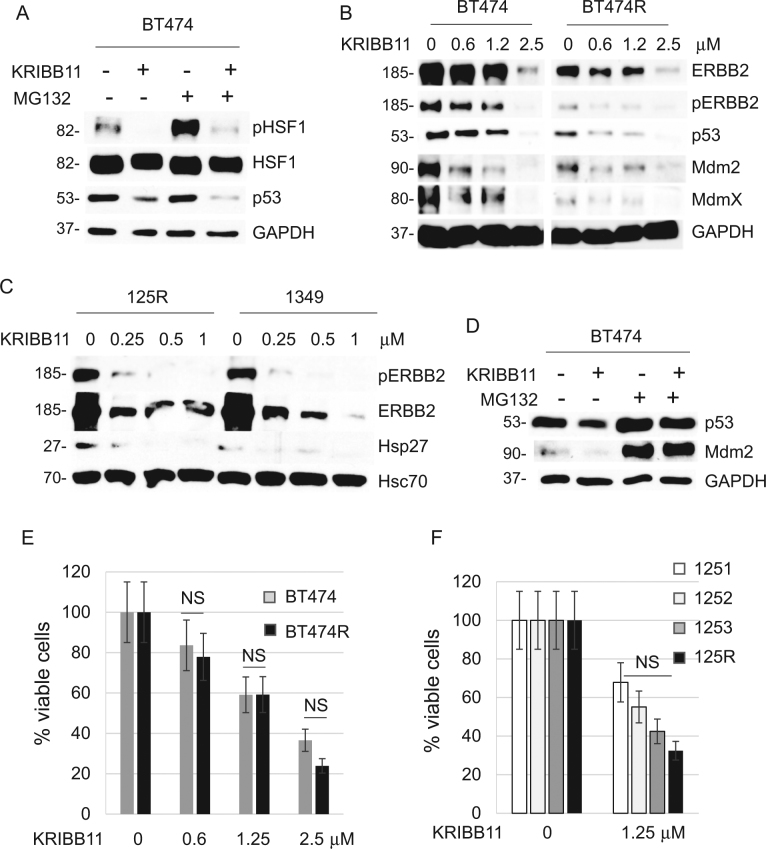
HSF1 inhibition causes degradation of mutp53 and ErbB2, and suppresses growth of both lapatinib-sensitive and lapatinib-resistant cancer cells. **a**–**d** The HSF1 inhibitor KRIBB11 suppresses activation of HSF1 (measured by phospho-Ser326) after MG132-induced proteotoxic stress (1 μM, 2.5 h) in lapatinib-sensitive BT474 cells (**a**), suppresses ERBB2 signaling and destabilizes mutp53 in both lapatinib-sensitive BT474 and lapatinib-resistant BT474R cells (**b**), suppresses ERBB2 signaling and HSF1 target Hsp27 in both lapatinib-sensitive 1349 and lapatinib-resistant 125R murine cells (**c**); and induces degradation of mutp53 and Mdm2 in lapatinib-sensitive BT474 cells, which is rescued by the proteasome inhibitor MG132 (**d**). Cells were pre-treated with KRIBB11 (2.5 μM, 24 h) followed by MG132 treatment (1 μM, 2.5 h) (**a**) or simultaneously treated with KRIBB11 (2.5 μM) and MG132 (2.5 μM) for 24 h (**d**). Western blot analyses, GAPDH and Hsc70 served as a loading control. **e**, **f** The HSF1 inhibitor KRIBB11 suppresses growth of human BT474R (**e**) and mouse 125R (**f**) lapatinib-resistant cells as efficiently as their corresponding lapatinib-sensitive controls, BT474 and 1251, 1252, 1349 cells, respectively. Cells were treated with indicated concentrations of KRIBB11 for 48 h, followed by cell viability assays, which are shown relative to DMSO-treated cells. One representative experiment out of two independent experiments (each performed in triplicate) is shown; NS non-significant

Therefore, the efficacy of Hsp90 inhibitors is limited by HSF1 function. Thus, we set to test the effect of specific HSF1 inhibitor KRIBB11 (N2-(1H-indazole-5-yl)-N6-methyl-3-nitropyridine-2,6-diamine)^21^ on lapatinib-resistant vs. lapatinib-sensitive cells. Consistently with a previous report^22^, KRIBB11 inhibits HSF1 phosphorylation with or without proteotoxic stress (MG132) (Fig. 5a). As a readout of HSP suppression, KRIBB11 also dose-dependently suppressed Hsp90 clients ERBB2, mutp53 and Mdm2 in both lapatinib-sensitive and lapatinib-resistant human BT474 and mouse 125R cancer cells (Fig. 5b, c). Similarly, to Hsp90 inhibition by ganetespib (Fig. 4d), KRIBB11 reactivated Mdm2 E3 ligase activity as manifested by downregulation of Mdm2 ubiquitination substrates MdmX, mutp53, and Mdm2 itself (Fig. 5b), which was rescued by the proteasome inhibitor MG132 (Fig. 5a, lanes 3, 4, Fig. 5d). These data indicate that HSF1 inhibition by KRIBB11 simultaneously targets both key oncogenic drivers, ERBB2 and mutp53, in lapatinib-sensitive and lapatinib-resistant ERBB2-overexpressing breast cancer cells. As a result, KRIBB11 dose-dependently kills lapatinib-sensitive and lapatinib-resistant human (Fig. 5e) and mouse (Fig. 5f) breast cancer cells with comparable efficiency.

### HSF1 inhibition suppresses adaptive RTK activation and overcome lapatinib resistance in ERBB2-positive breast cancer cells

**Fig. 6:**
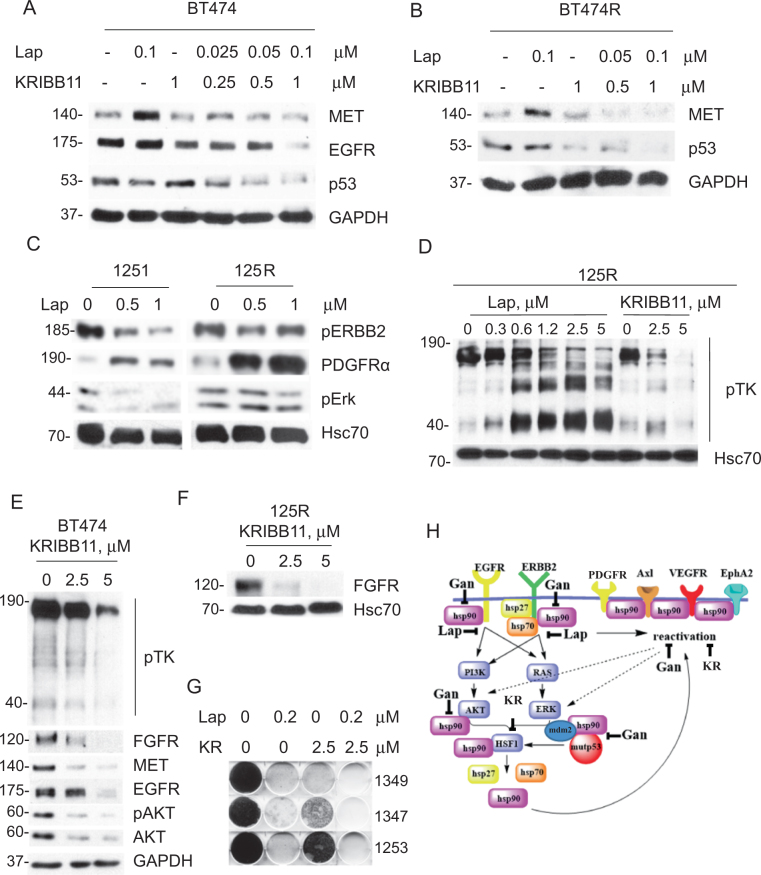
HSF1 inhibition bypasses lapatinib-induced adaptive signaling and prevents the onset of lapatinib resistance. **a**, **b** While lapatinib (0.1 µM, 48 h) upregulates MET in both lapatinib-sensitive BT474 (**a**) and lapatinib-resistant BT474R (**b**) cells, HSF1 inhibitor KRIBB11 reverses this effect. Moreover, KRIBB11 synergizes with lapatinib in degradation of EGFR and mutp53 (**a**) and restores responsiveness of mutp53 to lapatinib (**b**). Western blot analyses, GAPDH is a loading control. **c**–**f** Lapatinib (at indicated concentrations, 48 h) induces PDGFRα in both lapatinib-sensitive 1251 and lapatinib-resistant 125R murine cells (**c**) and induces global kinome activation (measured by phospho-Tyr antibody, pTK) in lapatinib-resistant 125R cells (**d**), KRIBB11 inhibits the global pTK activity and individual kinases in 125R cells (**d**, **e**), and BT474 cells (**f**). Western blot analysis, Hsc70 and GAPDH are loading controls. **g** KRIBB11 cooperates with lapatinib (at indicated concentrations, for 4 weeks) in suppressing emergence of lapatinib-resistant colonies. Colony formation assay. Representative images out of two technical replicas. **h** The proposed model. ERBB2 signaling mediates HSF1 activation^4,16^, which is potentiated by mutp53 via a feed-forward loop^5,15^, thereby upregulating Hsp90 clients including compensatory RTKs and mutp53 itself. Inhibition of ERBB2 by lapatinib leads to inhibition of HSF1 transcriptional activity and therefore decreased Hsp90 and release of Mdm2 from its inhibitory complex with Hsp90^4,5^ and subsequent degradation of mutp53 and Mdm2. KRIBB11 simultaneously inhibits diverse adaptive RTKs, as well as destabilizes potent oncogenic drivers—ERBB2, EGFR, and mutp53

Consistent with previous studies^3^, we noted a substantial heterogeneity of adaptive responses in lapatinib-resistant cancer cells, including RTKs such as MET^3^ and PDGFRα in human and mouse cells, respectively (Fig. 1). Interestingly, activation in response to lapatinib of both MET (Fig. 6a, b) and PDGFRα (Fig. 6c) occurred as quickly as 48 h after lapatinib treatment in lapatinib-resistant, as well as lapatinib-sensitive cells. It appears that it takes place at posttranscriptional level. RNAseg analysis of BT474 cells treated with lapatinib did not reveal induction of MET RNA transcript, while MET signaling was shown to be activated^3^. Since MET^22^ and PDGFRα^23^ are both Hsp90 clients, we asked if HSF1 inhibition by KRIBB11 would reverse MET and PDGFRα lapatinib-induced compensatory upregulation. Indeed, even the low KRIBB11 dose (1 µM, compare to Fig. 5b, e) alleviated lapatinib-induced MET upregulation in both lapatinib-sensitive and lapatinib-resistant BT474 cells (Fig. 6a, b). Moreover, KRIBB11 synergized with lapatinib in degrading mutp53 and EGFR in lapatinib-sensitive BT474 cells (Fig. 6a) and restored mutp53 responsiveness to lapatinib in lapatinib-resistant BT474R cells (Fig. 6b).

In addition, global assessment of Tyr-phosphorylated proteins—as an indirect readout of overall levels of kinases—revealed an extensive and dose-dependent kinome activation in response to lapatinib in murine 125R cells (Fig. 6d, left), while HSF1 inhibition by KRIBB11 suppressed global kinome activation (Fig. 6d, right) and individual Hsp90 kinase clients, e.g., ERBB2/pERBB2 (Fig. 5c), FGFR (Fig. 6e). To ensure that these effects are specific only to cancer cells, we tested the effect of lapatinib on normal mammary epithelial cells (MECs) isolated from H/H;ERBB2 mice. We found that lapatinib increases total pTK activity only in cancer, but not in MECs (Suppl. Fig. 1), suggesting cancer-specific mechanism of lapatinib-induced kinome reprogramming. This result is consistent with previous study showing high level and activity of HSF1 specifically in human tumor biopsies, but not in normal mammary tissues^24^. Also, global pTK signal and individual Hsp90 client kinases, e.g., ERBB2, pERBB2 (Fig. 5b), FGFR, MET, EGFR, AKT, and pAKT (Figs. 5b, 6f), were downregulated in human BT474 cells in response to HSF1 inhibition in a dose-dependent manner.

Finally, a colony formation assay showed that while lapatinib-sensitive murine 1349, 1347, and 1253 cells treated with lapatinib or KRIBB11 alone did develop resistant clones, the combinatorial lapatinib/KRIBB11 treatment completely blocked the emergence of resistance (Fig. 6g). Taken together, these results indicate that HSF1 inhibition suppresses global activation of compensatory RTK pathways in response to lapatinib, and therefore can prevent the onset of lapatinib resistance.

## Discussion

Although ERBB2-targeted therapies, such as lapatinib, revolutionized management of ERBB2-overexpressing breast cancer, primary and acquired resistance remains a major obstacle for the cure of this deadly disease. Therefore, understanding the mechanisms of lapatinib resistance will greatly facilitate development of successful combinatorial treatments with a durable therapeutic effect. In this study, we utilized an preclinical MMTV-ERBB2;mutp53 mouse model to investigate the mechanism of lapatinib resistance acquired in vivo in ERBB2-positive mammary tumors and to compare them to the resistance mechanisms acquired in vitro.

In both in vivo and in vitro scenarios, we found a robust kinome re-organization in response to ERBB2 inhibition by lapatinib. In agreement with previous studies^3^, a substantial heterogeneity of adaptive responses was observed in lapatinib-resistant cancer cells, including previously described (Axl, MET)^2^ and novel upregulated pathways, such as NFGR, MUSK, VEGFR1, PDGFRα, PDGFRβ, EPHA2, and EPHB2 (Fig. 1f). This multifaceted nature of compensatory responses underscores the difficulty of choosing the most effective drug combination to prevent or overcome lapatinib resistance. In this study we uncovered a common pro-survival mechanism of lapatinib resistance acquired in vivo and in vitro, i.e., an augmented HSF1-mediated heat shock response.

The oncogenic cooperation between ERBB2 and HSF1 was noted previously. Several in vivo studies demonstrated a crucial role of HSF1 in the development of ERBB2-driven breast cancer^10^. Not surprisingly, HSF1 protein levels are elevated in 80% of breast cancer cases that are associated with poor prognosis^25^. Although no clinical studies have directly analyzed the levels of HSF1 or HSPs in lapatinib-resistant tumors, emerging clinical evidence strongly supports our main conclusion. Thus, Phase II clinical trial with an Hsp90 inhibitor tanespimycin (17-AAG) plus trastuzumab (ERBB2-targeted therapy) showed a significant anticancer activity in patients with ERBB2-positive trastuzumab-resistant metastatic breast cancer^25^. Another a Phase I trial of ganetespib in combination with paclitaxel and trastuzumab in trastuzumab-refractory patients with human ERBB2-positive metastatic breast cancer showed significant clinical benefit of Hsp90 inhibition in triplet therapy^26^. Altogether, these clinical data strongly support the idea that inhibition of HSF1 and its downstream effectors (e.g., Hsp90) is effective strategy to overcome the resistance to ERBB2-targeted therapies.

Furthermore, we previously demonstrated that HSF1 is an important downstream effector of ERBB2 signaling and that lapatinib inhibits transcriptional activation of HSF1, by suppressing its Ser326 phosphorylation^5^. Most likely, lapatinib affects HSF1 function by inhibiting MAPK and AKT activation, both of which can induce transcriptional phospho-activation of HSF1 at Ser326^18, 19^. On the other hand, upregulation of compensatory RTKs in lapatinib-resistant cells can induce sustained MAPK and AKT signaling leading to enhanced S326-HSF1 phosphorylation and HSF1 protein stability. In support of this hypothesis we observed higher HSF1 protein and S326-HSF1 level after heat shock in lapatinib-resistant cells (Figs. 1g, h, 2e, f). Our previous study has shown that ERBB2 inhibition in lapatinib-sensitive cells impedes HSF1 activation, leading to the release of Mdm2 from its inhibitory complex with Hsp90 and to mutp53 destabilization^4^ (Figs. 3a, 6h). Strikingly, we now found that in lapatinib-resistant cells, lapatinib fails to modulate the ERBB2–HSF1–mutp53 axis (Fig. 3). Instead, HSF1 is constitutively activated and does not depend on the ERBB2 signaling (Fig. 3c), resulting in a superior tolerance of lapatinib-resistant cells to proteotoxic stress (Fig. 2).

We speculate that in lapatinib-resistant cells, the HSF1 function is restored by activation of adaptive RTKs and their downstream signaling components (Fig. 6h). In turn, sustained expression of HSPs promotes stability of their clients, adaptive RTKs, thus maintaining continuous HSF1 function (Fig. 6h). Although some elements of this model await further investigation, here we identified HSF1 as an upstream node of the lapatinib resistance mechanisms and demonstrated that its inhibition (i) suppresses global tyrosine-phosphorylation (Fig. 6d, f), (ii) alleviates lapatinib-induced upregulation of specific adaptive RTKs (Fig. 6a, b, c), (iii) synergizes with lapatinib in degradation of mutp53 (Fig. 6a, b), and (iv) prevents development of lapatinib resistance, as measured by appearance of lapatinib-resistant colonies (Fig. 6g).

Importantly, HSF1 inhibition in lapatinib-resistant cells restores mutp53 destabilization in response to lapatinib (Fig. 6b). Highly stabilized mutp53 levels are required for mutp53 oncogenic gain-off-function^17^, and mutp53 genetic and pharmacological ablation significantly suppresses malignant phenotypes in mutp53-carrying cancers^17^. Therefore, identification of compounds targeting mutp53 for degradation has a major translational impact for ERBB2-positive breast cancer therapy, given the high frequency of p53 mutations in this breast cancer subtype.

In sum, we showed that pharmacological inhibition of HSF1 simultaneously inhibits diverse adaptive responses endowing lapatinib resistance, as well as destabilizes potent oncogenic drivers of ERBB2-positive breast cancer, such as ERBB2, EGFR, and mutp53. Thus, targeting HSF1 and opens up a new therapeutic possibility for the clinical application of HSF1 inhibitors to prevent and/or delay onset of lapatinib resistance with a potential of the instant clinical translation.

## Materials and methods

### Human cancer cells

Human ERBB2-positive breast cancer cell line BT474 carrying E285K *TP53* mutation was purchased from ATCC in 2013. ATCC verifies cell’s identity with short tandem repeat analysis. To generate lapatinib-resistant BT474R cell line, parental BT474 cells were cultivated in the presence of increasing concentrations (100–300 nM) of lapatinib for 6 months, as previously described^3^. No further cell’s identity verification was performed. Unless indicated otherwise, lapatinib-resistant BT474R cells were routinely maintained in the presence of 300 nM lapatinib. Where shown, cells were treated with indicated concentrations of lapatinib (L-4899, LC Lab), MG132 (M7449, Sigma), ganetespib (STA-9090, Synta Pharmaceuticals, Lexington, MA, USA), KRIBB11 (385570, Calbiochem, Billerica, MA, USA). All cell viability assays were done using standard clonogenicity assays and CellTiter-Blue Cell Viability Assay (Promega, 96-well format with 5000 cells/well seeded 24 h prior). Prior to the CTB assay (Fig. 1a), cells were maintained in lapatinib-free media for 3 days. Cells were treated with drugs for 48 h, unless indicated otherwise, with drug concentrations as shown. Florescence was detected by SPECTRAmax M2 (Molecular Devices).

### Animals

MMTV-ERBB2 mice harboring activated ERBB2 were from Jackson Labs (strain FVBN-Tg(MMTV-ERBB2)NK1Mul/J). mutp53 R172H mice were a gift from G. Lozano^27^. Generation of R172H/+;ERBB2 compound mice was described previously^15^. Eight weeks old R172H/+;ERBB2 littermate females, all on C57Bl6/J:FVB/N 50:50 background, were treated with vehicle (18% Cremophor/3.6% dextrose) or lapatinib (75 mg/kg three times a week) by oral gavage lifelong. When lapatinib-treated tumors acquired lapatinib resistance, animals were treated with either vehicle, lapatinib alone, or lapatinib with ganetespib, as described in the text. Ganetespib was prepared as previously described^17^ and injected into the tail vein at 50 mg/kg once a week. At endpoint (tumor size ~3.5 cm^3^) mice were sacrificed and some of lapatinib only treated tumors were used to establish cell cultures. Mice were treated according to the guidelines approved by the Stony Brook University Institutional Animal Care and Use Committee.

### Establishing primary mammary tumor cell cultures

Mammary tumors were dissected from mice, rinsed three times in PBS, and sequentially digested with collagenase/hyaluronidase (37°C, 2 h), 0.05% Trypsin, DNAse I, and Dispase (Stem Cell Technology). The ensuing cell suspensions were treated with red blood cell lysis buffer, rinsed with PBS, resuspended in Opti-MEM medium (Gibco) and passed through a 40 µm mesh to remove cell chunks. Cells were plated on gelatin-coated plates and grown in CnT-BM1 medium (Cell-N-Tec). Unless indicated otherwise, lapatinib-resistant 125R cells derived from a lapatinib-resistant mammary tumor were routinely maintained in the presence of 300 nM of lapatinib. Heterozygous mutant p53 R172H/+ status was verified and confirmed by using genotyping primers^27^ in all established mouse cell lines.

### Immunoblot analysis and kinome arrays

For immunoblots, cell lysates with equal total protein content (2–20 µg) were blotted with antibodies to p53 (FL393), Mdm2, GAPDH, Hsc70 (all from Santa Cruz Biotechnology); Erk1, pErk1/2 (T202/Y204), EGFR, pEGFR (Y845), ERBB2, pERBB2 (Y1221/1222 and pY1248), MET, cleaved PARP, PDGFRα, PDGFRβ, FGFR, AKT, pAKT MdmX, pTK (all from Cell Signaling); HSF1, pHSF1 (S326), Hsp90, Hsp70, Hsp27 (all from Enzo Life Sciences Inc., Farmingdale, NY). All Western blots were repeated at least two times. The phospho-RTK array on primary mammary tumor cells was performed according to the manufacturer’s protocol (Mouse Phospho-RTK Array Kit, R&D Systems).

### Statistical analysis

Unpaired two-tailed Student’s *t*-test was used to calculate statistical significance (*p*-value). Kaplan–Meier analysis and log rank statistics were used to compare animal survival. All experiments were repeated in at least two biological replicas with three technical replicas each, unless indicated otherwise.

## Electronic supplementary material

Supplemental Figure 1

Supplemental Figure Legend
