# Supplementary figures and images for: Heat shock factor 1 confers resistance to lapatinib in ERBB2-positive breast cancer cells
Source: Cell Death Dis. 2018 May 24;9(6):621. doi: 10.1038/s41419-018-0691-x (PMC5967334; doi:10.1038/s41419-018-0691-x)

## Slide 1
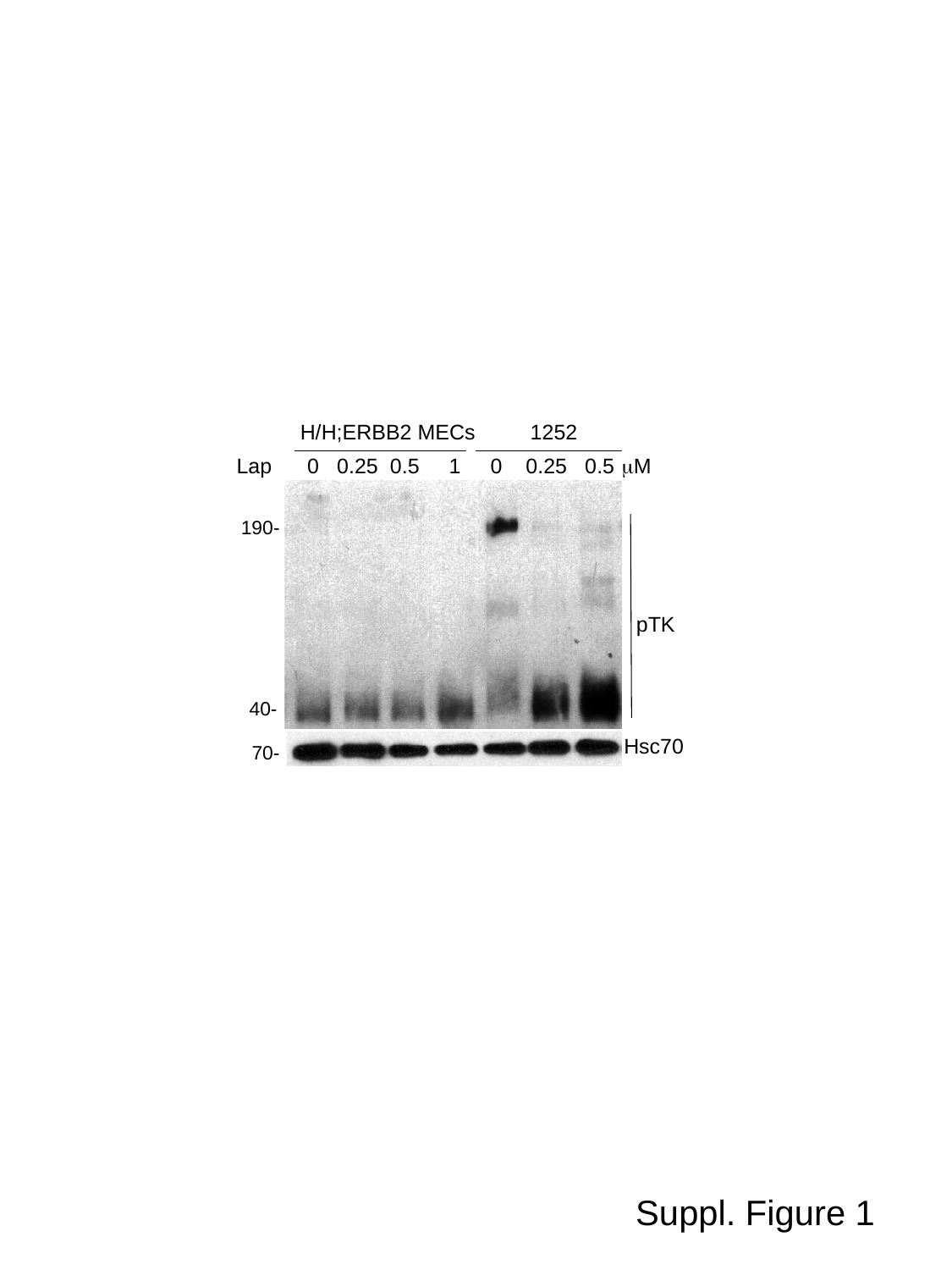

H/H;ERBB2 MECs
 1252
Lap 0 0.25 0.5 1 0 0.25 0.5 mM
190-
 pTK
40-
Hsc70
70-
Suppl. Figure 1

Supplement: Supplementary file 1 — Supplemental Figure 1 [file 41419_2018_691_MOESM1_ESM.pptx]
