# Supplementary material for: Heat shock factor 1 confers resistance to lapatinib in ERBB2-positive breast cancer cells
Source: Cell Death Dis. 2018 May 24;9(6):621. doi: 10.1038/s41419-018-0691-x (PMC5967334; doi:10.1038/s41419-018-0691-x)
Supplement: Supplementary file 2 — Supplemental Figure Legend [file 41419_2018_691_MOESM2_ESM.docx]

**Supplemental Figure 1.**

Lapatinib induces rapid kinome reprogramming indicated by the increase of global phospho-Tyrosine Kinase (pTK) activity in murine mammary tumor 1252 cells, but not normal mammary epithelial cells derived from H/H;ERBB2 mice, Hcs70 as a loading control.
